# Aberrant hiPSCs-Derived from Human Keratinocytes Differentiates into 3D Retinal Organoids that Acquire Mature Photoreceptors

**DOI:** 10.3390/cells8010036

**Published:** 2019-01-09

**Authors:** Rupendra Shrestha, Yao-Tseng Wen, Dah-Ching Ding, Rong-Kung Tsai

**Affiliations:** 1 Institute of Medical Sciences, Tzu Chi University, Hualien 970, Taiwan; dph.rupendra@gmail.com (R.S.); dah1003@yahoo.com.tw (D.-C.D.); 2 Institute of Eye Research, Hualien Tzu Chi General Hospital, Hualien 970, Taiwan; ytw193@gmail.com; 3 Department of Obstetrics and Gynecology, Hualien Tzu Chi General Hospital, Hualien 970, Taiwan

**Keywords:** keratinocytes, hiPSCs, genomic aberrations, retinal organoids, photoreceptors

## Abstract

Human induced pluripotent stem cell (hiPSC)-derived three-dimensional retinal organoids are a new platform for studying the organoidogenesis. However, recurrent genomic aberration, acquired during generation of hiPSCs, limit its biomedical application and/or aberrant hiPSCs has not been evaluated for generation of differentiated derivatives, such as organoids and retinal pigment epithelium (RPE). In this study, we efficiently differentiated mosaic hiPSCs into retinal organoids containing mature photoreceptors. The feeder-free hiPSCs were generated from the human epidermal keratinocytes that were rapid in process with improved efficiency over several passages and maintained pluripotency. But, hiPSCs were cytogenetically mosaic with normal and abnormal karyotypes, while copy number variation analysis revealed the loss of chromosome 8q. Despite this abnormality, the stepwise differentiation of hiPSCs to form retinal organoids was autonomous and led to neuronal lamination. Furthermore, the use of a Notch inhibitor, DAPT, at an early timepoint from days 29–42 of culture improved the specification of the retinal neuron and the use of retinoic acid at days 70–120 led to the maturation of photoreceptors. hiPSC-derived retinal organoids acquired all subtypes of photoreceptors, such as *RHODOPSIN*, *B-OPSIN* and *R/G-OPSIN*. Additionally, the advanced maturation of photoreceptors was observed, revealing the development of specific sensory cilia and the formation of the outer-segment disc. This report is the first to show that hiPSCs with abnormal chromosomal content are permissive to the generation of three-dimensional retinal organoids.

## 1. Introduction

Human induced pluripotent stem cells (hiPSCs), an alternative to embryonic stem cells, provide new possibilities for studying in vitro organoidogenesis, disease modeling, gene correction strategies, patient-specific drug screening and autologous cell transplantation [1,2,3,4]. hiPSC technology is based on the basic principle of converting differentiated somatic cells into undifferentiated pluripotent stem cells by the overexpression of defined transcription factors (Oct4, Sox2, c-Myc and Klf4) [5]. In recent years, iPSCs have been extensively used for the replacement of lost photoreceptors in animal model with retinal degeneration (preclinical trials) [6,7,8,9] and clinical trials using autologous transplantation of hiPSC-derived retinal pigment epithelium (RPE) in patients with age-related macular degeneration [10]. However, due to induced tumorigenesis associated with oncogenes used in iPSC generation and recurrent genomic aberrations acquired during reprogramming, the use of iPSCs for clinical applications is limited [11,12,13]. Also, there is the possibility of culture-induced chromosomal aneuploidies and copy number variations in hiPSCs over several passages [14,15]. Despite certain drawbacks of hiPSCs, there is rapid progress in minimizing the possibilities of viral integration, transgenes and associated tumorigenesis [16,17]. Additionally, the generation of functional 3-D organoids that closely mimic the naïve organs or tissues has led to an in vitro 3-D complex model with physiological relevance. These organoids systems have greater advantages in the engineering of human organs, such as the eye, brain, heart, gut, liver, lung and stomach, among others [3].

Remarkable progress has been made in the differentiation of cell-specific lineages using hiPSCs as alternative approaches for generating retinal cells, such as retinal pigment epithelium (RPE), photoreceptors (PRP) and retinal ganglion cells (RGC) [18,19,20]. Additionally, hiPSC-derived 3-D retinal organoids (also known as retinospheres, miniretinas, optic cups, optic vesicles and 3-D retinal tissues) that accurately recapitulate the retinogenesis, mimic in vivo biological parameters of the human retina [20,21,22,23,24,25,26]. Three-dimensional retinal organoids offer potential sources of various specialized retinal neurons, such as photoreceptor precursors (CD73^+^), that can be isolated for transplantation in an animal model with photoreceptor degeneration [6]. Recently, published data has revealed the improved visual functions in rats with retinal degeneration using a sheet of hESC-derived retinal organoids [27]. Therefore, retinal organoids are an exciting model that can be used to prepare organoid sheets containing various retinal progenitors and facilitate the in vitro production of photoreceptors for preclinical transplantation. This evidence supports the tremendous importance of retinal organoids in the translational research and the progression toward clinical application. Our basic goal was to generate 3-D retinal organoids in vitro by modulating differentiation parameters to acquire mature photoreceptors. In addition, chromosomal abnormalities are commonly associated with cell transformation (somatic cells to hiPSCs) but their impact on the differentiation of retinal organoids has not been evaluated. This insight has led us to examine whether aberrant hiPSCs contribute to the differentiation of retinal organoids and recapitulate retinal neurogenesis.

In this study, we generated feeder-free hiPSCs from human keratinocytes that acquired recurrent chromosomal abnormalities and copy number variations at a higher passage number (passage 16). Despite these genetic abnormalities, the hiPSCs showed a positive contribution and efficiently differentiated into retinal organoids. Additionally, the organoids recapitulated retinal neurogenesis and acquired mature photoreceptors, including rod and cone cells. Although the differentiation of hiPSCs to retinal organoids was autonomous, DAPT and retinoic acid supplementation at a defined timepoint improved neurogenesis and enhanced the maturation of photoreceptors.

## 2. Material and Methods

### 2.1. NHEK Primary Cell Line and Growth Conditions

The adult, single-donor, cryopreserved, normal human epidermal keratinocytes (NHEK) primary cell line was purchased from PromoCell GmbH, Heidelberg, Germany (cat. No. C-12003). Briefly, the NHEK cell line was maintained in precoated plates containing EpiLife medium (Invitrogen, Carlsbad, CA, USA) and EpiLife human keratinocytes growth supplement (HKGS) (Invitrogen) at 37 °C in a 5% CO_2_ incubator. The precoating of plates was performed using 1 mL of premixed coating matrix (recombinant human type I collagen) (Invitrogen, Carlsbad, CA, USA) diluted in medium and incubated for 30 min at room temperature. The medium was changed every 48 h and subcultured once 75% confluency was obtained. For storage, the cell line was prepared in freezing medium (1:10 DMSO:FBS) with a density of 1 × 10^7^ cells per mL in each cryovial. The cryovials were placed in pre-cool ‘Mr Frosty’ freezing unit at −80 °C for overnight and transferred to liquid nitrogen for storage.

### 2.2. Generation of Feeder-Free hiPSCs from Human Keratinocytes

A feeder-free hiPSC line was generated from human keratinocytes with a nonintegrating system using CytoTune™-iPS 2.0 Sendai reprogramming kit (Invitrogen, Carlsbad, CA, USA, cat. No. A16517). The system used Sendai particles to deliver defined transcription factors that included three vector preparations as follows: polycistronic Klf4–Oct3/4–Sox2, cMyc and Klf4. For this procedure, NHEK cells were seeded in precoated plates at a density of 5 × 10^4^ cells per well (34.8 mm diameter) in EpiLife medium without antibiotics. The medium was changed every other day to obtain healthier cells and 30–40% cell confluency with small clusters of 5–6 keratinocytes at approximately 2–3 days. Then, the cells were transduced with a cocktail of three vector preparations at an MOI of 4:4:2, Klf4–Oct3/4–Sox2:cMyc:Klf4 (company recommended ratio is 5:5:3 but not able to observe hiPSC colonies, we changed the ratio as per manufacturer’s suggestion) in the prewarmed EpiLife medium. At 24 h post infection, the cells were washed with 1X Dulbecco’s PBS (-Ca^2+^/Mg^2+^) (Gibco, Massachusetts, United States) and fed with fresh EpiLife medium containing HKGS. At 7 days post infection, the cells were passaged with TrypLETM Express (Gibco, Massachusetts, United States) onto vitronectin (rhVTN-N) (Gibco, Massachusetts, United States)-coated 6-well plates at a density of 2 × 104 cells/well and fed every day with feeder-free chemically defined Essential 8 TM medium (Gibco, Massachusetts, United States) and 10 μM Y-27632 (Merck Millipore Massachusetts, United States). At 10–12 days post-transduction, live cell staining was performed either with Tra-1-60 or Tra-1-81 and positive hiPSC colonies were manually isolated, passaged using the EDTA method [28] (0.5 M EDTA, pH 8.0, Invitrogen, Carlsbad, CA, USA) and clonally expanded on fresh vitronectin-coated plates. The cells were cultured in standard growth conditions at 37 °C in a 5% CO_2_ incubator.

### 2.3. Genomic Stability of hiPSCs

The genomic stability of hiPSCs was assessed by G-banded karyotyping to analyze mosaicism and iCS-digital^TM^ Pluri test to determine structural aberrations and recurrent genomic abnormalities. Passage 16 hiPSCs were used to assess genomic stability, as described below.

#### 2.3.1. G-Banded Karyotyping

The human iPSC line was karyotyped using a standard G-banding protocol at the Cytogenetics Lab, Center of Medical Genetics, Hualien Tzu Chi Hospital. Briefly, the overnight cultured cells were treated with 10 μg/mL of Colcemid at 37 °C for 45 min and the cells were then harvested by trypsinization, swollen with 0.54% KCl solution and fixed with Carnoy’s fixative. Then, a metaphase spread was prepared by dropping the fixed swollen cells onto a microscopic glass slide. Once the metaphase spread dried, the chromosomes were stained using Giemsa stain and analyzed using a ZEISS Axio Z2 microscope. The karyotype analysis was performed using an Ikaros karyotyping system (MetaSystems Group, Inc., Boston, MA, USA) and at least 12 metaphases were karyotyped.

#### 2.3.2. Digital PCR: Copy Number Variation (CNV)

The human iPSC line was submitted to the Stem Genomics—Bio-incubateur Cyborg—IRMB—Hôpital Saint Eloi, France, to detect recurrent genomic abnormalities. Briefly, DNA was extracted from the hiPSCs and an iCS-digital^TM^ Pluri test was performed using 24 specific probes. Digital PCR was used to detect the copy number variation in target chromosomes.

### 2.4. In Vitro Differentiation of hiPSCs into Embryoid Bodies (EBs)

In vitro formation of EBs from hiPSCs is an important alternative method to in vivo teratoma assays. For this procedure, hiPSC colonies were dissociated with 0.5 mM EDTA into small clumps of 2–4 hiPSC cells. The dissociated cells were cultured with chemically defined Essential 8^TM^ medium and 10 μM Y-27632 in ultralow adhesion 6-well plates (Corning, Lowell, MA, USA). On the following day, small aggregates of hiPSCs were observed and fed with fresh Essential 8^TM^ medium every 2 days without Y-27632. The EB-like aggregates were maintained for different time points (7, 14 and 28 days). EBs in suspension culture were maintained in standard growth conditions at 37 °C in a 5% CO_2_ incubator.

### 2.5. In Vitro Differentiation of hiPSCs: Early Stages to 3-D Retinal Organoids

Retinal differentiation was achieved with a slight modification of a previously published protocol [24]. The pluripotency of hiPSCs was maintained by culturing hiPSCs in the chemically defined feeder-free Essential 8^TM^ medium (E8M). Briefly, to initiate differentiation, the hiPSCs were dissociated into small clumps and cultured in suspension to induce the formation of EBs, as described above. The EB suspension culture was maintained for 5 days and then induced to the retinal cell fate using neural induction medium (NIM) containing DMEM/F12 (1:1) (Gibco), 1% N2 supplement (Gibco), 1X minimum essential media non-essential amino acids (NEAA) (Gibco) and 2 μg/mL heparin (Stem Cell Technologies). Gradient neural induction was performed starting at day 5 using the following combinatorial medium formulations: E8M:NIM (3:1) on day 5, E8M:NIM (1:1) on day 6 and E8M:NIM (1:3) on day 7, followed by 100% NIM on day 8. The EBs were cultured until day 10 in suspension, after which the EBs were plated at a density of 10–15 EBs per cm^2^ onto vitronectin-coated plates containing NIM. Next, from day 16–35, the cultures were fed every other day with fresh retinal differentiation medium (RDM) containing DMEM/F12 (3:1), 2% B27 (Gibco), 1X NEAA (Gibco), 1X sodium pyruvate (Gibco), 0.1 M β-mercaptoethanol (Gibco), 1% knockout replacement serum (Gibco) and 1% penicillin/streptomycin (Gibco). During differentiation, the morphologically identical optic cup and bipotent retinal progenitor cells were manually isolated at day 28 and day 35 respectively, under a cell imaging microscope (Invitrogen, EVOS XL Core) in a culture hood using fine-pointed surgical needles. The cells were collected and cultured in ultralow adhesion plates containing RDM and gradually formed 3-D retinal organoids. For long-term culture, the RDM was supplemented with 10% fetal bovine serum, 1% N2 (Gibco, Grand Island, NY, USA), 100 μM taurine (Sigma, St. Louis, MO, USA) and 1X GlutaMAX (Gibco). In addition,10 μM DAPT (Millipore, Merck KGaA, Darmstadt, Germany) was supplemented from days 29 to 42. For the maturation of photoreceptors, 500 nM all-trans-retinoic acid (Sigma) was added from days 70 to 120. During retinal differentiation, the cultures were maintained in standard growth conditions at 37 °C in a 5% CO_2_ incubator. The media components were slightly modified and additional DAPT was used, which differed from the previously published recipe. Retinal organoids with intact 3-D structures and smooth peripheral boundaries were selected at different weekly timepoints include in weeks (W4, W6, W7, W8, W13, W18 and W20) for the analysis of retinal neurogenesis.

### 2.6. Immunostaining and Immunofluorescence Analysis

Indirect immunofluorescence live cell staining of primary hiPSCs were performed with Tra-1-60 and Tra-1-81 for identifying the reprogrammed colonies. Briefly, the medium was removed from reprogramming dish and rinsed gently once with 1X knockout DMEM/F12. The cells were incubated for 1 h with primary antibodies at 37 °C. After incubation, the dish was rinsed three times with 1X knockout DMEM/F12. Then, the cells were incubated with Alexa Fluor-conjugated secondary antibodies for 1 h at 37 °C and then washed three times with 1X knockout DMEM/F12. Finally, the cells were covered with 2 mL of fresh knockout DMEM/F12 and microscopy was performed. All the wash medium and antibodies used in live cell staining was maintained sterile to avoid contamination for the subculture.

Immunofluorescence analysis was performed on hiPSCs or differentiated cells grown on chamber slides (EMD Millipore^TM^ EZ Slides) and cryosectioned retinal organoids. Briefly, adherent cells and organoids were fixed in 4% paraformaldehyde (PFA, Sigma) for 20 and 45 min, respectively, at room temperature. The fixed retinal organoids were subjected to dehydration with successive gradient sucrose concentrations as follows: 10% for 30 min, 20% for 60 min and 30% for overnight successively. The samples were embedded in OCT compound (Tissue-Tek, Sakura, Torrance, CA, USA), frozen and stored at −80 °C until use. Thin sections of 15 μm slices were cut using a cryostat microtome (Leica CM-3050-S Cryostat) and collected on SuperFrost glass slides. The slides were washed twice with 1X PBS and incubated in blocking solution (1.5% bovine serum albumin, 0.05% gelatin, 0.25% Triton X 100, 0.025% Tween 20 and 0.025% sodium azide) for 45 min at room temperature. Following blocking, the slides were treated with specific primary antibodies at the manufacturer’s recommended dilutions and incubated at 4 °C for overnight. On the following day, the slides were rinsed three times with 1X PBS and treated with Alexa Fluor-conjugated secondary antibodies for 1 h in the dark at room temperature. The slides were then rinsed three times in 1X PBS, the cell nuclei were counterstained cell nuclei with DAPI (4′,6-diamidino-2-phenylindole) (Sigma Aldrich, St. Louis, MO, USA) for 10 min and the slides were coverslipped using DPX mounting medium (Sigma Aldrich, St. Louis, MO, USA). Fluorescence images were acquired using an A1^+^ confocal microscope (Nikon). The primary and secondary antibodies that were used are listed in Table 1.

### 2.7. RNA Extraction and Reverse-Transcription PCR

The PCR was performed for EB-specific markers at 7, 14 and 28 days while hiPSCs marker at different passage, such as P4, P6, P8, P10 and P12. Briefly, total RNA extraction was performed in duplicate using a PureLink^TM^ RNA Mini kit (Invitrogen) according to the manufacturer’s recommended protocol. The quality of RNA was evaluated by calculating the ratio of the absorbance at 260 nm versus 280 nm that was obtained using a Nanodrop spectrophotometer (NanoDrop ND-1000, Wilmington, DE, USA) and samples with the ratio of 1.8–2.2 were subsequently used. One microgram of total RNA was used to synthesize cDNA using an iScript^TM^ cDNA synthesis kit (Bio-Rad, Foster, CA, USA). Standard reverse-transcription PCR was performed using OnePCR reaction mixture (GeneDireX) with the following optimized PCR conditions of 38 cycles: 94 °C × 40 s for denaturation, 53–56 °C × 1 min for annealing and 72 °C × 2 min for extension. The PCR products were analyzed using 2% agarose gels. The primers that were used are listed in Table 2.

### 2.8. TEM Ultrastructural Analysis

hiPSC-derived retinal organoids were submitted to the Electron Microscope Laboratory at Tzu Chi University for ultrastructural analysis using their standard protocol. Briefly, the organoids were fixed in a cold EM fixative (2.5% glutaraldehyde/0.1 M cacodylate buffer + 1% tannic acid) overnight at 4 °C. Then, the organoids were postfixed with 1% osmium tetroxide/0.1 M cacodylate buffer, stained with 2% aqueous uranyl acetate (en bloc staining), dehydrated and embedded in Spurr’s resin. Ultrathin sections of 80 nm slices were cut using an ultramicrotome (Leica EM UC6, Hernalser Hauptstrasse, Vienna, Austria), collected on formvar-coated single-slot grids and examined under a transmission electron microscope (TEM, H-7500, Hitachi High-technologies, Tokyo, Japan) at 80 kV. The photoreceptor-specific sensory cilia and their compartments were investigated for week 20 retinal organoids.

## 3. Results

### 3.1. Rapid and Efficient Generation of Feeder-Free hiPSCs from Human Keratinocytes

**Figure 1:**
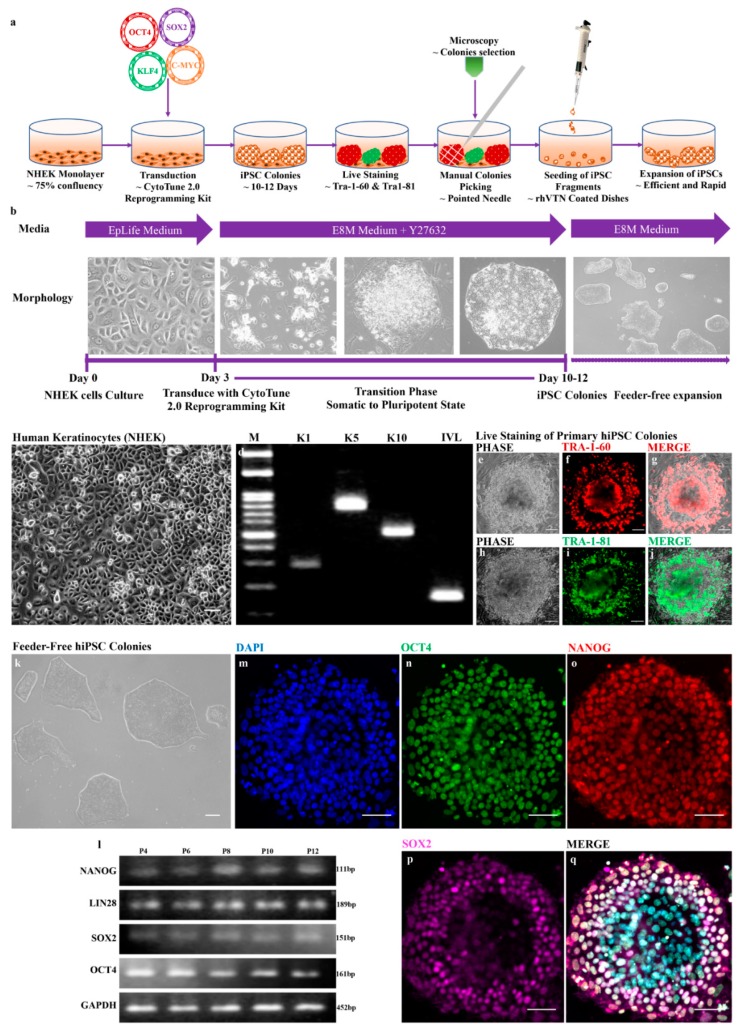
hiPSCs derived from normal human epidermal keratinocytes (NHEKs) exhibit pluripotency. (**a**) Schematic illustration of hiPSC generation; (**b**) Timeline showing the generation of hiPSCs from NHEK cells; (**c**) Monolayer culture of human keratinocytes (NHEK); and (**d**) reverse-transcription PCR analysis showing the expression of keratinocyte markers, such as keratin 1 (*K1*), keratin 5 (*K5*), keratin 10 (*K10*) and involucrin (*IVL*); (**e**–**j**) live staining of primary hiPSC colonies; (**k**) Feeder-free hiPSC colonies; (**l**) reverse-transcription-PCR analysis showing the consistent expression of pluripotency markers, such as *OCT4*, *SOX2*, *LIN28* and *NANOG* and the internal control (*GAPDH*) at different passages (P4, P6, P8, P10 and P12); and (**m**–**q**) immunocytochemistry staining of hiPSCs (P4) showing expression of pluripotency markers, such as *OCT4*, *SOX2* and *NANOG*. Scale bars, 100 μm (**c**,**k**); 150 μm (**e**–**j**) 590 μm (**m**–**q**).

hiPSCs can be generated from various types of differentiated cells but with different efficiencies. To generate hiPSCs, we used normal human epidermal keratinocytes (NHEKs) that were cryopreserved at passage 2 from PromoCell. The cells were cultured in EpiLife medium and maintained their morphologically identical rounded and cobblestone appearance (Figure 1c). To validate that the obtained cells were keratinocytes, we performed reverse-transcription PCR that showed the expression of keratinocyte-specific markers, such as keratin 1 (*K1*), keratin 5 (*K5*), keratin 10 (*K10*) and involucrin (*IVL*) (Figure 1d). Furthermore, passage 4 or 5 cells were used for the reprogramming experiments. The illustrated protocols and timelines for reprogramming are shown in Figure 1a,b, respectively. We generated feeder-free hiPSCs from human keratinocytes in vitronectin-coated dishes using Essential 8 medium. Importantly, we showed rapid hiPSCs colony formation within 10–12 days and double repeats of the reprogramming experiments showed similar rapidity, in agreement with previous reports [29]. In a single transduction experiment, approximately 35–40 hiPSC primary colonies were observed at a cell seeding density of 2 × 10^4^ cells per well in a 6-well plate. All the primary hiPSCs were positive for Tra-1-60 and Tra-1-81 screened using live cell staining (Figure 1e–j). The use of actively dividing cells in standard culture conditions showed the improved reprogramming of human keratinocytes to hiPSCs compared with cells used after long-term freezing in liquid nitrogen. Additionally, the expansion of feeder-free hiPSCs improved over several passages (up to passage 16) and their pluripotency was maintained. hiPSC colonies maintained their round morphology with well-defined sharp edges/boundaries and contained tightly packed cells that were similar to the ESC-like phenotype (Figure 1k). Passage 4 hiPSCs were used for the immunofluorescence analysis to reveal the expression of pluripotency markers, such as *OCT4*, *SOX2* and *NANOG* (Figure 1m–q). Additionally, the improved expansion with the identical morphology of hiPSCs over several passages was analyzed for the expression of pluripotency markers using reverse-transcription PCR (rt-PCR). The results showed the consistent expression of makers, such as *OCT4*, *SOX2*, *NANOG* and *LIN28*, at different passages (P4, P6, P8, P10 and P12) of the hiPSCs (Figure 1l). Our data revealed that the generation of hiPSCs from human keratinocytes is rapid, efficient and maintains stemness in feeder-free culture conditions.

### 3.2. Recurrent Chromosomal Abnormalities and Copy Number Variation in hiPSCs

**Figure 2:**
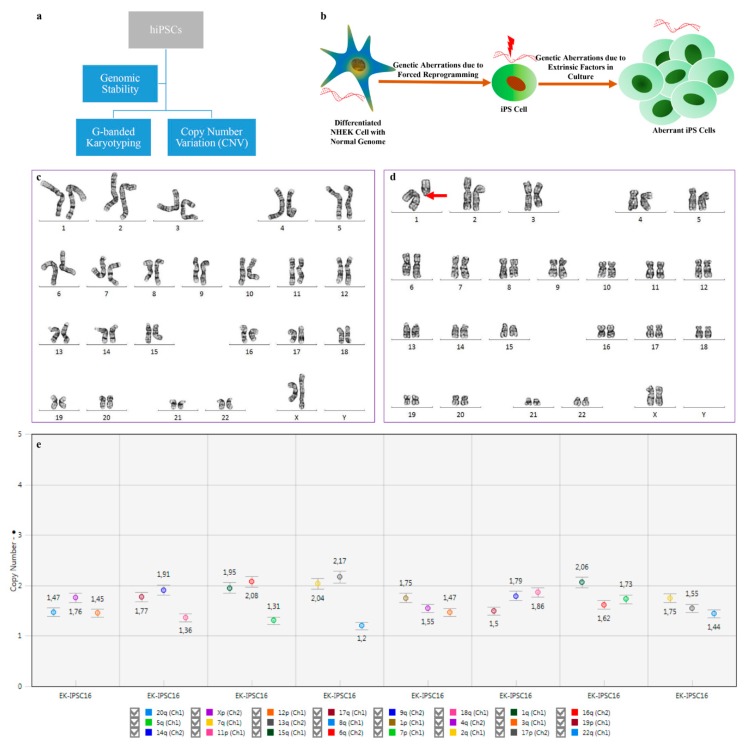
Prolonged culture of hiPSCs causes several genetic alterations that impact applications due to functional consequences. (**a**) Flowchart highlighting the methods for analyzing genomic stability; (**b**) Illustration showing the factors associated with genomic aberrations; (**c**) Cytogenetic analysis showing of normal (46,XX) and (**d**) abnormal (46,XX,del(1)(q11)[cp2]) karyotypes; (**e**) Graph showing the copy number values for 24 probes targeted for specific chromosomes. The legend should be read from the left to the right and line by line.

The analysis of genomic integrity is routinely implemented for the characterization of hiPSCs. In this study, we analyzed cytogenetic aberrations and the copy number variation (CNV) in passage 16 hiPSCs using G-banded karyotyping and digital PCR, respectively, as shown in Figure 2a. hiPSC-associated genomic aberrations are linked with forced reprogramming/selective pressure and/or culture adaptation during in vitro maintenance [30]. A schematic illustration of genomic instability is shown in Figure 2b. In this study, we observed that hiPSCs were mosaic with normal and abnormal karyotypes in the 12 metaphases that were analyzed. Out of the 12 metaphases, 6 metaphases revealed a normal karyotype, while the other 6 metaphases included deletions, losses and chromosomal rearrangements. The major chromosomal imbalance observed was a loss of chromosomes 4, 7, 11 and 14, while some cells acquired unbalanced structural abnormalities of chromosomes 1 and 9 that showed deletions, such as del(1)(q11)[cp2], del(9)(q11) and derivatives of 9 with unknown translocation. The recurrent chromosomal abnormalities and respective karyotypes of each analyzed are shown in Table 3. The mosaic hiPSC cell line with a normal diploid 46,XX karyotype (Figure 2c) and abnormal 46,XX,del(1)(q11)[cp2] karyotype (Figure 2d) are the representative images. Therefore, our results suggest that the hiPSCs line was karyotypically abnormal due to long-term in vitro maintenance. The hiPSCs might have had a random probability distribution that cannot be predicted precisely with each metaphase due to mosaicism.

Because G-banded karyotyping can detect chromosomal aneuploidies and genomic aberrations larger than ~5 Mb, we used digital PCR to identify a small percentage of copy number variations (CNVs). The results revealed the loss of chromosome 8q and a few other chromosomes, indicating a trend toward loss. The CNVs ranging in criteria of loss, trend to loss, gain, trend to gain and normal were based on the copy number values, as shown in Table 4. The copy number value with each target chromosome is shown in the graph (Figure 2e), which shows the loss of chromosome 8q. Our data suggest that cytogenetic abnormalities did not show the similarity with copy number variations, possibly owing to the mosaicism and the difference in the size-dependent detection limit. Overall, we report that hiPSCs are prone to *de novo* chromosomal aberrations and CNVs either due to forced reprogramming/selective pressure or due to long-term cultural adaptation.

### 3.3. Self-Forming Embryoid Bodies (EBs) from Feeder-Free hiPSCs

**Figure 3:**
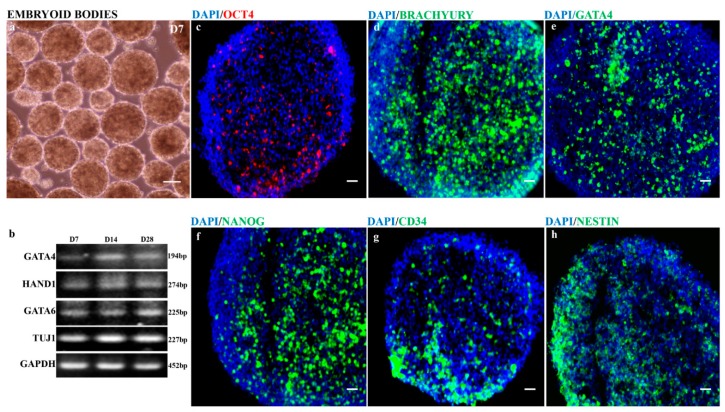
hiPSC-derived free-floating embryoid bodies (EBs) exhibit germ layers. (**a**) Suspension culture of embryoid bodies at day 7; (**b**) reverse-transcription PCR analysis revealed the consistent expression of mesoderm (*HAND1* and *GATA6*), endoderm (*GATA4*), ectoderm (*TUJ1*), & internal control (*GAPDH*) markers for at different stages of EBs (days 7, 14 and 28); and (**c**–**h**) immunohistochemistry staining of cryosectioned EBs at day 7 showed the expression of pluripotency (*OCT4* and *NANOG*), mesoderm (*BRACHYURY* and *CD34*), endoderm (*GATA4*) and ectoderm (*NESTIN*) markers. Scale bars, 50 μm (**c**–**h**); 100 μm (**a**).

The in vitro differentiation of hiPSCs into EBs is an important alternative to in vivo teratoma assays to demonstrate the pluripotency and can be used as a standard functional assay [31]. To assess the pluripotency of keratinocyte-derived hiPSCs, we performed a simple suspension method to generate human EBs that are an amalgam of the three developmental germ layers (ectoderm, mesoderm and endoderm). We report that the formation of human EBs did not require antidifferentiation factors. The feeder-free hiPSCs in suspension culture formed EBs and differentiated into multicellular three-dimensional aggregates in a highly autonomous manner. hiPSC-derived EBs at day 7 were round and heterogeneous in size (Figure 3a) and were cut into a thin section of 12–15 μm slices and collected on SuperFrost glass slides. The immunofluorescence analysis of cryosectioned EBs showed the expression of pluripotency (*OCT4* and *NANOG*), mesoderm (*BRACHYURY* and *CD34*), endoderm (*GATA4*) and ectoderm (*NESTIN*) markers (Figure 3c–h). Additionally, EBs were maintained for different timepoints (days 7, 14 and 28) to assess the differentiation status and identify cell-specific genes using reverse transcription-PCR. The human EBs showed the expression of cell-specific genes for mesoderm (*HAND1* and *GATA6*), endoderm (*GATA4*) and ectoderm (*TUJ1*) markers (Figure 3b). However, the differentiation status was consistent throughout for all three different timepoints and showed a similar pattern of cell-specific gene expression (Figure 3b). Therefore, our data suggest that hiPSCs have the self-forming ability to produce three-dimensional human EBs and differentiate into the lineages of germ layers without the need for antidifferentiation factors. Human EBs that recapitulate many aspects of the developmental process during the embryogenesis can also be used to drive to retinal organoids.

### 3.4. hiPSC-derived EBs Differentiated into Primitive Anterior Neuroepithelium and Early Neural Retinas

**Figure 4:**
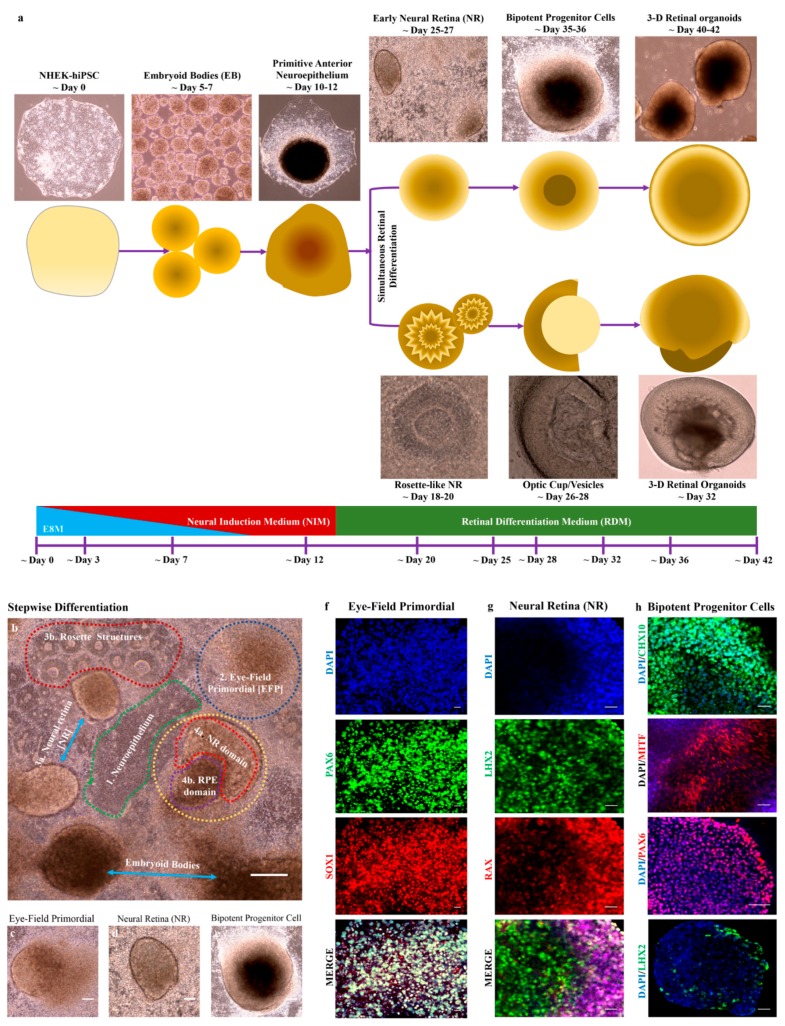
hiPSC-derived 3-D embryoid bodies differentiated into retinal progenitors that were self-organized into eye-field primordial cells and formed neural retina (NR) cells and bipotent retinal progenitor cells. (**a**) Schematic illustration showing the generation of retinal organoids either via bipotent retinal progenitor cells or the optic cup/vesicles system; (**b**) Differentiation of EBs in a stepwise manner of early eye development; (**c**–**e**) 2-D cell culture with representative image of eye field primordial cells, neural retina (NR) cells and bipotent retinal progenitor cells; and (**f**–**h**) immunocytochemistry staining the expressing eye field primordial cells (*PAX6* and *SOX1*) (**f**); neural retina cells (*LHX2* and *RAX*) (**g**); & bipotent progenitor cells (*CHX10*, *MITF*, *PAX6* and *LHX2*) (**h**). Scale bars, 50 μm (**c**–**e**); 100 μm (**f**,**g**,**h**); 200 μm (**b**).

The in vivo development of the retina beginning from the embryonic neural plate has been mapped with the formation of the eye-field. In this study, we differentiated hiPSCs to mimic the early stage of eye development. We revealed that during the analogous differentiation of the retina in vitro, the formation of 3-D retinal organoids was achieved either by the formation of bipotent retinal progenitor cells or the optic cup/vesicles (Figure 4a). After induction with NIM for 7 days on adherent culture plates, hiPSC-derived EBs underwent differentiation, as shown in Figure 4b. The differentiation was initially achieved by the formation of a flat uniform primitive anterior neuroepithelium and a centrally and tightly packed domain called the eye field primordial (EFP) (Figure 4c). The anterior neuroepithelium underwent differentiation to form rosette structures and some of this structure formed rosette-like neural retina capable of forming optic cups. Additionally, the EFP domain formed simultaneously during culture and differentiated into the early neural retina (NR) (Figure 4d). NR cells were morphologically round with sharp edge boundaries and contained centrally packed retinal progenitor cells. The NR cells further differentiated to form bipotent retinal progenitor cells (NR domain and RPE domains) (Figure 4e). The representative stages of early eye development, such as eye field primordial, neural retina (rosette-like NR or early neural retina) and bipotent retinal progenitor cells, were observed with typical morphologies (Figure 4c–e). These cells showed the expression of stage-specific markers that were analyzed by immunohistochemistry. The eye-field primordial cells were positive for *PAX6* and *SOX2* (Figure 4f), the neural retina cells expressed *LHX2* and *RAX* (Figure 4g) and the bipotent retinal progenitor cells showed the expression of *MITF*, *CHX10*, *PAX6* and *LHX2* (Figure 4h). However, the expression of LHX2 in bipotent cells was low.

### 3.5. hiPSC-derived Retinal Organoids

**Figure 5:**
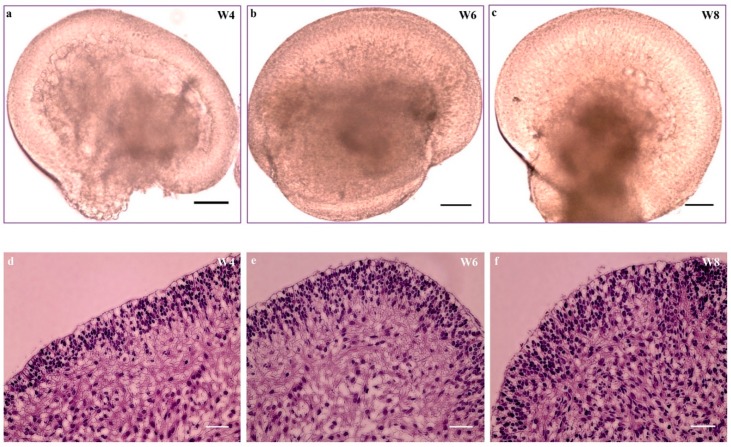
Hematoxylin and eosin staining of retinal organoids derived from hiPSCs. (**a**–**c**) Retinal organoids and (**d**–**f**) H&E staining of cryosectioned organoids at week 4 (**d**); week 6 (**e**); and week 8 (**f**). Scale bar, 200 μm (**a**–**f**).

The simultaneously formed optic cup/vesicles (days 26–28) or bipotent retinal progenitor cells (days 35–36) in monolayer culture were morphologically identical. These cells were manually isolated, collected and cultured in a free-floating suspension system. The identical cells in suspension acquired the 3-D structure of retinal organoids (Figure 5a–c). The organoids comprised morphologically thick and transparent NR domains with adjacent rolled-up bulges of retinal pigment epithelium (RPE). The NR domain includes pseudostratified epithelium with proliferating cells that spontaneously differentiate following the characteristics of retinal neurogenesis [24]. Histologically, the organoids showed a distinct apical layer and a varying density of cells from the center to the periphery of the organoids at different stages (weeks 4, 6 and 8) (Figure 5c–f). The organoids with progenitor cells at week 4 began to differentiate, increased cell density with specific cell types and migrated to their appropriate retinal layers.

### 3.6. hiPSC-derived Organoids with Retinal Cell Specification and Laminated Structures

**Figure 6:**
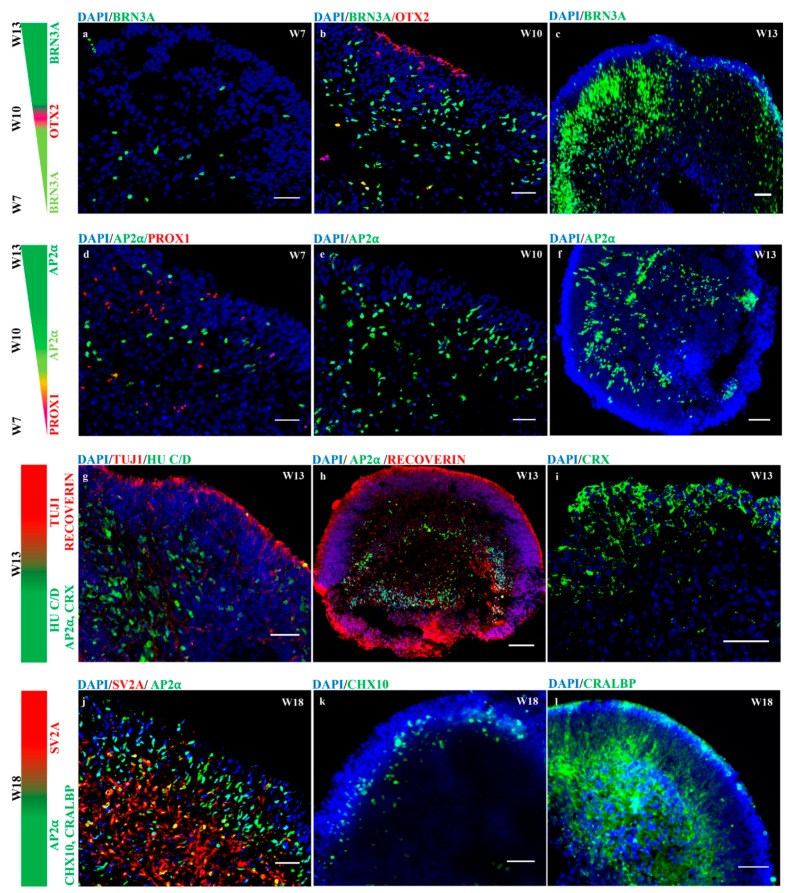
hiPSC-derived 3-D retinal organoids recapitulate retinal neurogenesis. Retinal progenitor cells (RPC) within 3-D retinal organoids differentiated into specific retinal neurons and organized themselves in the proper layers at specific timepoints; retinal *BRN3A*-positive retinal ganglion cells, (**a**–**c**), *AP2α*-positive amacrine cells (**d**–**f**,**h**,**j**), precursors for major neuronal cells (ganglion and amacrine cells) positive for *HU C/D* (**g**), *OTX2-*, *RECOVERIN-*, & *CRX*-positive photoreceptor precursor cells (**b**,**h**,**i**), *PROX1*-positive horizontal cells (**d**), *CHX10*-positive bipolar cells (**k**), *TUJ1*-positive nerve fiber layer (**g**), *SV2A*-positive outer plexiform layer (**j**) and *CRALBP*-positive Müller cells (**l**). The colors indicate for representative makers and intensities at different timepoints. Scale bars, 50 μm (**d**,**i**); 100 μm (**b**,**c**,**f**–**h**,**j**–**l**); 200 μm (**a**,**e**).

In vivo neurogenesis in the vertebrate neural retina begins with the differentiation of multipotent retinal progenitor cells (RPCs). RPCs determine the retinal fate (neurons or glial cells) in response to the patterning signals. These signals stimulate retinal neurogenesis that follows the fixed temporal sequences, in which retinal ganglion cells (RGCs) and horizontal cells (HCs) differentiate first, followed by cone photoreceptors, amacrine cells (ACs), rod photoreceptors, bipolar cells (BCs) and finally, Müller cells (MCs) [32,33]. This specific neuronal and glial cell assembly in the neuroretina is essential for proper visual. In this study, we report in vitro retinal neurogenesis in organoids generated from keratinocyte-derived hiPSCs. To achieve retinal organoidogenesis, the cultures were supplemented with fetal bovine serum, taurine and DAPT for 42 days. Furthermore, retinoic acid (RA) was supplemented from days 70 to 120 to achieve the maturation of photoreceptors. The organoids in long-term culture maintained their regular round shape, steady growth and improved survival in low attachment 6-well plates. Approximately 10–12 organoids/well were maintained for a long-term by changing the media every 2 days. Immunofluorescence analysis revealed the retinal neurogenesis in individual organoids grown at a different timepoints and indicated the approximate time that specific neuronal markers began to be expressed. Notably, retinal ganglion cells, horizontal cells and amacrine cells were first observed at week 7, expressing *BRN3A*, *PROX1* and *AP2α*, respectively (Figure 6a,d). Along with retinal ganglion and amacrine cells, photoreceptor progenitor cells positive for the *OTX2* transcription factors were observed at week 10 and populated the apical zone (developing outer nuclear layer) (Figure 6b). The *BRN3A-* and *AP2α*-positive cells densities increased over time, migrated and arranged themselves in an appropriate layer at week 13 in the basal and intermediate zones of the neural retina (Figure 6a–f,h). Additionally, retinal organoids at week 13 showed distinct layers of neuronal cells (retinal ganglion and amacrine cells) that expressed *HU C/D* in the baso-intermediate zone (Figure 6g). The developing nerve fiber-like layers positive for *TUJ1* at week 13 appeared in a wave-like fashion from the intermediate to the apical zones (Figure 6g). Furthermore, a distinct layer of photoreceptors expressing *RECOVERIN* and *CRX* populating the apical zone was observed at week 13 (Figure 6h,i). As the time progressed, a well-organized outer nuclear layer in retinal organoids was observed and developed an outer plexiform layer that expressed synaptic vesicle protein markers (*SV2A*) at week 18 (Figure 6j). In addition, developing layers of bipolar cells positive for *CHX10* and Müller’s cells expressing *CRALBP* were observed at week 18 and populated in their appropriate layers (Figure 6k,l). Interestingly, the amacrine cells expressing *AP2α* were observed until week 18 (Figure 6j). Our data suggest that retinal organoids generated from keratinocyte-derived hiPSCs revealed the efficient retinal neurogenesis with a supplementation of DAPT and RA. The organoidogenesis experiment was conducted with 2 replicates at a similar condition where at least 6–8 organoids were analyzed and revealed consistent results for retinogenesis.

### 3.7. Acquisition of Mature Photoreceptors Expressing Highly Differentiated Rods and Cones Markers

**Figure 7:**
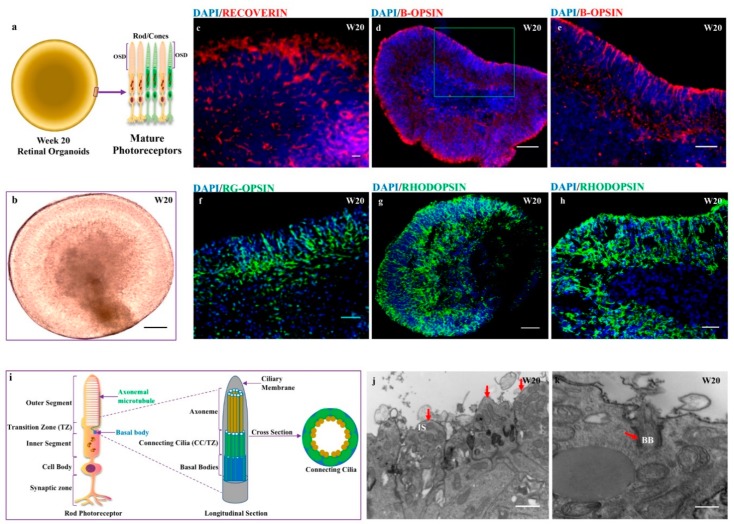

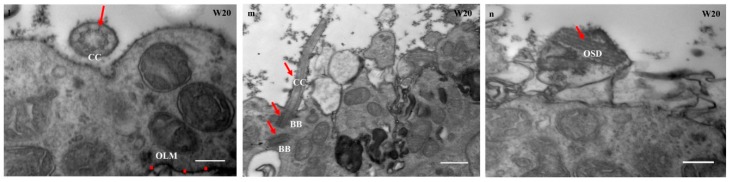
hiPSC-derived 3-D retinal organoids in long-term culture acquire mature photoreceptors with the formation of the outer-segment disc. (**a**) Illustration of week 20 retinal organoids showing mature photoreceptors (rod/cones) with stacks of the outer-segment disc (OSD); (**b**) Representative images of retinal organoids; (**c**) Immunohistochemistry staining of cryosectioned organoids expressing *RECOVERIN*; (**d**,**e**) and *B-OPSIN*; (**e**) Enlarged part of image d is shown in green box in image d (**f**) *RG-OPSIN*; and (**g**,**h**) *RHODOPSIN* markers; (**i**) Illustration showing longitudinal and cross sections of rod photoreceptors; and (**j**–**n**) transmission electron microscopy (TEM) analysis of week 20 organoids shows the presence of distinct compartments of the sensory cilia-like inner segment (IS) of the photoreceptors (**j**); basal bodies (BB) with protruding cilia (**k**); cross section of connecting cilia (CC) and outer limiting membrane (OLM) (**l**); long extended axoneme of connecting cilia with basal bodies (**m**); and stacks of the outer segment disc packed with rhodopsin (**n**). The functional domains with red arrows and cross marks in the respective images. Scale bars, 50 μm (**f**); 100 μm (**c**–**e**); 200 μm (**b**,**g**,**h**,**j**–**n**).

Several protocols revealing the evidence for robust and efficient generation of laminated retinal organoids from pluripotent stem cells have been reviewed extensively [34]. However, the generation of retinal organoids with highly differentiated rods and cones requires and efficient strategy. The use of retinoic acid (RA) [24,35,36] and the Notch signaling inhibitor DAPT [26,36] has shown a greater yield of highly differentiated photoreceptors. However, the mature photoreceptors containing all photoreceptor subtypes have been generated in the niche of retinal organoids derived from urine-hiPSCs without supplementation of retinoic acids [23]. In this study, we further revealed that the use of DAPT until day 42 and supplementation of retinoic acid from days 70–120 enriched the generation of all photoreceptor subtypes by week 20. hiPSC-derived organoids with *RECOVERIN-* and *CRX*-positive photoreceptors at week 13 became comparably dense cells at the apical zone expressing markers for photoreceptors from week 20. The photoreceptors with rods and cones cells were dominantly arranged in the apical zone of organoids (Figure 7a,b). Immunofluorescent analysis revealed the greater expression of immature photoreceptor marker *RECOVERIN* at week 20 (Figure 7c). Additionally, rods and cones were detected in the appropriate layer of the apical zone with proper distribution at 20 weeks of differentiation. Immunostaining of mature photoreceptors showed the expressions of *B-OPSIN*, *R/G-OPSIN* and *RHODOPSIN* markers at week 20 (Figure 7d–g) and a more polarized distribution of *RHODOPSIN* was acquired in some organoids at week 20 (Figure 7h). By week 20, 70% of the organoids (n-7/10) showed a consistent distribution of rods and cones. Our data revealed that retinal organoids generated from keratinocyte-derived hiPSCs were rich in *B-OPSIN-*, *R/G-OPSIN-* and *RHODOPSIN*-specific cells. Furthermore, the ultrastructural analysis of retinal organoids at week 20 showed a distinct compartment of photoreceptor-specific sensory cilia (Figure 7i). Transmission electron microscopy of randomly selected 2 retinal organoids at week 20 showed the inner and outer segment structures (Figure 7j,l). Maturation of the photoreceptors was evident with the presence of sensory cilia, including centriole basal bodies with protruding cilia and long extending axonemal-connected cilia (Figure 7k,m), with a cross section of connecting cilia (9 × 2 + 0) (Figure 7l) and intracellular membrane discs reminiscent of outer-segment discs in native photoreceptors (Figure 7n). Overall, we showed that photoreceptor-enriched retinal organoids were generated from keratinocyte-hiPSCs and achieved a high degree of maturation containing sensory cilia as a functional domain.

## 4. Discussion

In this study, we generated the feeder-free hiPSCs from normal human epidermal keratinocytes and further differentiated the hiPSCs into 3-D organoids with retinal cell specification and neuronal lamination. However, the hiPSCs acquired recurrent chromosomal and genomic aberrations that might be due to forced reprogramming, selective pressure and cultural adaptation. Despite the abnormal karyotype and copy number variation, the expansion of hiPSCs improved at higher passage numbers and became efficient at consistently expressing pluripotency markers at different passages and were able to differentiate into the developmental germ layers. Additionally, hiPSC-derived retinal organoids that recapitulate retinogenesis mimicked the in vivo development of the human retina. Retinal neurons acquired in the organoids were arranged in their proper zones with supplementation of DAPT and RA at a defined timepoints. Importantly, hiPSC-derived photoreceptors were arranged in the apical zone of the organoids and achieved advanced maturation, demonstrating specific sensory cilia and stacks of the outer segment disc.

In recent years, iPSCs have substantially impacted biomedical research and have become a promising alternative to embryonic stem cells (ESCs), which have moral objections and are ethically controversial [37]. In general, iPSCs are generated with the established protocol of reprogramming somatic cells by overexpressing four defined transcription factors (Oct4, Sox2, c-Myc and Klf4) [5,38,39,40]. This type of cell includes derivatives of developmental germ layers, such as mesodermal fibroblasts, epithelial cells of endoderm origin and ectodermal keratinocytes [5,41,42,43]. Considering that retinal cell specifications begin from the ectoderm, we used human epidermal keratinocytes as the somatic cell source to generate hiPSCs. In this study, we generated feeder-free hiPSCs in vitronectin coated plates with a simple approach using Essential 8 medium and ROCK inhibitor. Our feeder-free and normoxic culture conditions maintained the classical morphology similar to ESCs; small homogenous cells with low a N:C (nucleus: cytoplasm) ratio and organized flat colonies with defined borders. hiPSC generation was a rapid process with ESC-like colonies observed in 10–12 days and an improved efficiency was observed at higher passages, consistent with other reports [29,44]. The higher reprogramming efficiency of keratinocytes might be due to the ectodermal in origin that avoids the mesenchymal to epithelial transition (MET) and the high-level expression of endogenous cMyc and Klf4 [45,46]. However, our keratinocyte-derived hiPSC lines at passage 16 contained chromosomal aberrations. The hiPSCs revealed mosaic cell lines with normal diploid and abnormal karyotypes [30]. The major abnormalities included the loss of chromosomes 4, 7, 11 and 14 and an imbalance in structural abnormalities in chromosome 1 and 9. Furthermore, CNV analysis of the same passage revealed the loss of only chromosome 8q. It has been suggested that the chromosomal aberrations accumulate later at high passage number while CNVs are lost [14,15]. However, these aberrations are permissive to hiPSC differentiation into embryoid bodies and retinal organoids with laminated structures. However, the possibility of organoid formation might be due to flexibility of genome during differentiation or due to clonal selection of normal hiPSCs. The genomic integrity in the organoids require further investigation before preclinical and clinical study. Furthermore, using keratinocytes as ectoderm derivatives to generate hiPSC proved to offer greater potential with the capability of self-forming retinal organoids with retinal cell types.

First, hiPSCs were differentiated into embryoid bodies using ROCK inhibitors in suspension culture. The formation of EBs in suspension culture with reduced homogeneity might be due to existing small clumps of dissociated hiPSCs and newly formed aggregates. However, the use of ROCK inhibitor is not an essential factor for EB formation [47] but is recommended for improving cell survival on the first day of suspension culture [48]. hiPSC-derived EBs are an amalgam of developmental germ layers capable of differentiating into specific cell types. We report that the neural induction of EBs has the potential to differentiate into all major stages of the early eye and retina. All the stages were morphologically identical, while the expression of *PAX6* and *RAX* resembled a primitive stage of the eye field in human retina development. The formation of the eye field begins from the primitive anterior neuroepithelium and the expression of *PAX6/RAX* markers identify the anterior neuroepithelium [36,49]. It was evident that the formation of either bipotent retinal progenitor cells and optic vesicles began with the differentiation of the eye field. Bipotent cells are positive for both *MITF* and *CHX10*, modulating either RPE or neuroretinal fate [50,51]. However, the expression of *CHX10* is predominant in optic vesicles as an early marker of neural retina progenitors but acquired a mature neuronal phenotype [51]. Our data demonstrated that keratinocyte-derived hiPSCs differentiated in a highly autonomous manner with the spatiotemporal expression of *PAX6* and *RAX* in the eye field, while *MITF* and *CHX10* were expressed in bipotent cells. These results support that hiPSCs progressed through the early stages of eye development to form retinal tissues.

After retinal fate specification, bipotent retinal progenitor cells or optic vesicles in 3-D culture expressed early retinal markers, which led to mature retinal cell types. In this study, we demonstrated that hiPSCs differentiated into retinal organoids in 3-D culture without any extrinsic signaling factors and underwent retinogenesis and neuronal lamination that mimicked the native human retina. The development of 3-D retinal organoids or tissues has been optimized with variations in all published protocols arguing whether to use exogenous factors or not. However, the common theme is that pluripotent stem cell-derived retinal organoids contain all major retinal cell types arranged in their appropriate layers and become well-organized laminated structures with photoreceptors in the apical zone and retinal ganglion cells in the intermediate zone [20,24,52,53,54,55,56]. In this study, we used fetal bovine serum for the long-term maintenance of cell survival, taurine for retinal neurons and DAPT and RA for the maturation of photoreceptors in an optimized manner that was modified from a previously published protocol [24,36,55,57]. However, the continued use of retinoic acid in culture delays the maturation of photoreceptors and retinal pigment epithelium [55,58]. In addition, the use of Notch signaling inhibitors in an early period of retinal fate specification can change retinal progenitors to neurogenic and photoreceptor lineages [55,57]. Based on these observations, we used DAPT until day 42 and retinoic acid (RA) between days 70–120 in longitudinal cultures of retinal organoids. Similar to a previous report, the use of DAPT for 12–14 days accelerated the differentiation of retinal progenitors to the neurogenic fate and improved the noticeable formation of *CRX-* and *RECOVERIN*-positive photoreceptors at week 13. Along with the use of DAPT until day 42, the addition of RA between days 70–120 showed the presence of *RG-OPSIN-* and *B-OPSIN*-positive cones and *RHODOPSIN*-positive rod photoreceptors. Overall, we suggest that the use of DAPT at early stages of retinal organoids promotes the differentiation of progenitors into photoreceptors that form specific rods and cones photoreceptors in the presence of retinoic acids.

The advanced level maturation of the photoreceptors, including the presence of specific sensory cilia and their distinct compartmentalization (observed with transmission electron microscopy), was evident from our data, suggesting that this might be due to the implementation of DAPT and RA and the use of keratinocytes as ectodermal derivatives, such as the retina. The formation of retinal organoids with progenitor cells was highly autonomous but DAPT supplementation was required for the early neurogenic fate specification and RA was required for photoreceptor maturation, which is inconsistent with previously reported data [23]. Fibroblasts are commonly used somatic cells for the generation of retinal organoids with a spatiotemporal pattern of retinogenesis in retinal organoids [22,24,26]. Similar to the retina, we used ectodermal derivative-keratinocytes as the somatic cell source to generate hiPSCs. The hiPSCs were further differentiated into retinal organoids that showed neuronal lamination and achieved all subtypes of photoreceptors within week 20, which suggests that using ectodermal derivatives as a source for hiPSC reprogramming enables the efficient differentiation to retinal organoids that are rich in rod and cone cells. Our data showed more efficient expression of *RHODOPSIN* at week 20 than a previous report that revealed the less rod opsin expression in retinal organoids generated from keratinocyte-derived hiPSCs at week 25 [24]. A possible explanation for the higher efficiency of *RHODOPSIN* expression in the present study is that DAPT supplementation enhanced the generation of photoreceptor progenitors to facilitate the photoreceptor development at an early stage. Additionally, our study provides evidence that genomic aberrations in hiPSCs are permissive to the differentiation of retinal organoids with retinal neurogenesis. A contribution of retinogenesis in organoids could be cell-line dependent, the effect of exogenous factors, culture conditions and technical handling. In addition to numerously published protocols, variations persist in the retinal fate specification, the time of differentiation and the mechanism regulating the differentiation of photoreceptor subtypes. Therefore, this indicates the further validation and experimentation is required to generate retinal organoids with all types of retinal neurons arranged in their proper layers in single organoids.

## 5. Conclusions

Our results demonstrated that ectodermal keratinocytes were rapidly reprogrammed into hiPSCs in feeder-free and normoxic conditions. However, chromosomal aneuploidies were acquired in the keratinocyte-derived hiPSCs. The hiPSCs were successfully differentiated into retinal organoids in our in vitro culture system with little or no impact of loss chromosomal 8q. It is evident that considerable flexibility exists in the genome that are compatible with organoid generation from mosaic hiPSCs. Thus, our data provides important clue that organoid generated from abnormal hiPSCs can be used for understanding disease mechanism.

## Figures and Tables

**Table 1:** List of the primary and secondary antibodies that were used in this study.

Target	Brand-Catalogue Number	Dilution	Host
**Primary Antibodies**			
Tra-1-60	Invitrogen; 41–1000	1:100	Mouse
Tra-1-81	Invitrogen; 41–1100	1:100	Mouse
OCT4	Abcam; ab27985	1:200	Goat
Alexa Fluor 647 anti-SOX2	BioLegend; 656108	1:200	Mouse
NANOG	Millipore; AB9220	1:200	Rabbit
BRACHYURY	Abcam; AB20680	1:200	Rabbit
CD34	Abcam; ab81289	1:100	Rabbit
GATA4	Millipore; AB4132	1:200	Rabbit
NESTIN	Sigma; N5413	1:100	Rabbit
PAX6	Millipore; AB2237	1:100	Rabbit
SOX1	Abcam; ab87775	1:500	Rabbit
LHX2	Santa Cruz Biotechnology; SC19344	1:50	Goat
RX	Novus Biologicals; H00030062-M02	1:200	Mouse
MITF	Abcam; ab80651	1:200	Mouse
CHX10	Novus Biologicals; NBP184476	1:1000	Rabbit
BRN3A	Millipore; MAB1585	1:20	Mouse
OTX2	R&D; MAB1979	1:50	Mouse
AP2α	Invitrogen; MA1872	1:50	Mouse
PROX1	Millipore; AB5475	1:2000	Rabbit
TUJ1	BioLegend; PRB-435P	1:1000	Rabbit
HU C/D	Invitrogen; A21271	1:100	Mouse
CRX	Abnova; H00001406-M06	1:200	Mouse
RECOVERIN	Millipore; AB5585	1:1000	Rabbit
SV2A	Invitrogen, PA5-52476	1:50	Rabbit
CRALBP	Abcam; ab15051	1:200	Mouse
B-OPSIN	Millipore; AB5407	1:200	Rabbit
RG-OPSIN	Millipore; AB5405	1:200	Rabbit
RHODOPSIN	Abcam; ab5417	1:1000	Mouse
**Secondary Antibodies**			
Alexa Fluor 488 Anti-Goat	Invitrogen; A11055	1:500	Donkey
Alexa Fluor 555 Anti-Goat	Invitrogen; A21432	1:500	Donkey
Alexa Fluor 647 Anti-Goat	Invitrogen; A21447	1:500	Donkey
Alexa Fluor 488 Anti-Mouse	Invitrogen; A21202	1:500	Donkey
Alexa Fluor 555 Anti-Mouse	Invitrogen; A31570	1:500	Donkey
Alexa Fluor 647 Anti-Mouse	Invitrogen; A31571	1:500	Donkey
Alexa Fluor 488 Anti-Rabbit	Invitrogen; A21206	1:500	Donkey
Alexa Fluor 555 Anti-Rabbit	Invitrogen; A31572	1:500	Donkey
Alexa Fluor 647 Anti-Rabbit	Invitrogen; A31573	1:500	Donkey

**Table 2:** List of primer sequences for specific genes.

Genes	Forward Primers (5′-3′)	Reverse Primers (5′-3′)	Size (bp)
**Human Keratinocytes-Specific Markers**			
*IVL*	CTGCCCACAAAGGGAGAAGT	AGCGGACCCGAAATAAGTGG	166
*K1*	CCCTCCTGGTGGCATACAAG	GTTGGTCCACTCTCCTTCGG	293
*K10*	CCCTGGGCTAAACAGCATCA	TCCAGAGCCCGAACTTTGTC	525
*K5*	TCAACAAGCGTACCACTGCT	CTGCTACCTCCGGCAAGAC	802
**hiPSC-Specific Markers**			
*OCT4*	CAGTGCCCGAAACCCACAC	GGAGACCCAGCAGCCTCAAA	161
*SOX2*	GGGAAATGGGAGGGGTGCAAAAGAGG	TTGCGTGAGTGTGGATGGGAT TGGTG	151
*NANOG*	CAGAAGGCCTCAGCACCTAC	ATTGTTCCAGGTCTGGTTGC	111
*LIN28*	TGCACCAGAGTAAGCTGCAC	CTCCTTTTGATCTGCGCTTC	189
**Embryoid Body-Specific Markers**			
*GATA4*	TCCCTCTTCCCTCCTCAAAT	TCAGCGTGTAAAGGCATCTG	194
*HAND1*	TGCCTGAGAAAGAGAACCAG	ATGGCAGGATGAACAAACAC	274
*GATA6*	CCTCACTCCACTCGTGTCTGC	GTCCTGGCTTCTGGAAGTGG	225
*TUJ1*	CAGAGCAAGAACAGCAGCTACTT	GTGAACTCCATCTCGTCCATGCCCTC	227
*GAPDH*	ACCACAGTCCATGCCATCAC	TCCACCACCCTGTTGCTGTA	452

**Table 3:** Cytogenetic analysis of hiPSCs with chromosomal aberrations.

Type of Abnormality	Cytogenetic Analysis	Metaphase
Normal	46,XX	6
Deletion	46,XX,del(1)(q11)[cp2]	1
Deletion	46,XX,del(9)(q11)	1
Loss	44,XX,-7,-11	1
Loss	45,XX,-4	1
Loss	45,XX,-14	1
Derivative, Translocation and Loss	46,XX,+der(9)t(9;？)(p13;？),-11	1
Status: Abnormal	Karyotype: 46,XX,del(1)(q11)[cp2]∕46,XX [6]	Total: 12

**Table 4:** Copy number variations in hiPSCs with the loss of chromosome 8q.

Category	Copy Number Values	Chromosomes
Loss	1.3	8q
Trend to loss	1.5	3q, 12p, 18q, 19p, 20q, 22q
Normal	2.0	1p, 1q, 2q, 4q, 4q, 6q, 7p, 7q, 9q, 11p, 13q, 14q, 15q, 16q, 17p, 17q,
Trend to gain	2.5	-
Gain	2.7	-
